# A prospective observational study correlating possible novel biomarkers with disease severity and antihistamine response in chronic spontaneous urticaria

**DOI:** 10.5415/apallergy.0000000000000132

**Published:** 2024-01-11

**Authors:** Divya Bhatia, Hitaishi Mehta, Anuradha Bishnoi, Niharika Srivastava, Keshavamurthy Vinay, Davinder Parsad, Muthu Sendhil Kumaran

**Affiliations:** Department of Dermatology, Venereology and Leprology, Post Graduate Institute of Medical Education and Research, Chandigarh, India.

**Keywords:** Apolipoprotein, biomarkers, chronic spontaneous urticaria, complement, immunoglobulin E, interleukin-9

## Abstract

**Background:**

Role of complement fraction 5a (C5a), interleukin (IL)-9, and apolipoprotein (apo) A-IV as biomarkers of disease severity and antihistamine response in chronic spontaneous urticaria (CSU) remains elusive.

**Objective:**

To identify the role of C5a, IL-9, and apo A-IV as potential biomarkers in predicting disease severity and antihistamine response in CSU patients.

**Methods:**

This was a prospective observational study of 95 patients and 42 controls. Serum analysis of C5a, IL-9, and apo A-IV was done using enyzme linked immunosorbent assay kits. Also, serum IgE and anti-thyroid peroxidase (TPO) levels were assessed in all patients. All patients were started on oral levocetirizine 5 mg at baseline and dose was titrated upwards to maximum of 20 mg based on response. Patients were categorized into antihistamine responders or nonresponders as per their disease response. Serological markers, serum IgE, and anti-TPO were correlated with baseline disease severity and antihistamine response.

**Results:**

C5a levels were significantly higher in cases as compared to controls (*P* = 0.004). Significantly higher IL-9 levels were observed in antihistamine responders than nonresponders (*P* = 0.008). Baseline urticaria severity demonstrated a statistically significant positive and negative correlations with IL-9 (*ρ* = 0.277, *P* = 0.007) and apo A-IV (*ρ* = −0.271, *P* = 0.008) levels, respectively. Levels of serum IgE (*P* = 0.031) and anti-TPO (*P* = 0.039) were significantly higher in antihistamine nonresponders compared to responders.

**Conclusions:**

IL-9 and apo A-IV might be potential novel biomarkers to predict urticaria severity. Higher IL-9 might be a predictor of antihistamine response. Elevated anti-TPO and serum IgE might predict poor antihistamine response.

## 1. Introduction

Chronic spontaneous urticaria (CSU) is characterized by the presence of wheals with or without angioedema, which occurs daily or almost daily for more than 6 weeks [1]. Mechanisms reported to have a probable role in the pathogenesis include autoimmunity and autoallergy [2], complement pathway [3], coagulation pathway [3], and chronic infection [4]. Role of complement pathway in triggering mast cell degranulation and in augmentation of autoimmune pathway is well known [3, 5, 6]. Although higher complement fraction 5a (C5a) serum levels have been documented in chronic urticaria, data on its role as a biomarker predicting disease severity and antihistamine refractoriness are limited [3]. A crucial role of interleukin (IL)-9 has been described in various atopic disorders, autoimmune inflammation, and antitumor immunity in mice models [7–9]. Its preliminary role has been predicted in urticaria due to markedly elevated levels of Th9 cells observed in peripheral blood by few authors [10]. Due to a positive correlation between Th9 cells and IL-9, it has been postulated that CSU patients might have a higher IL-9 expression in peripheral blood. Role of IL-9 in atopic dermatitis is well elucidated; however, regarding its role in chronic urticaria, only a few reports are available [11–13]. Apolipoprotein (apo) A-IV is an antiinflammatory molecule having role in lipid transport, antiatherogenic pathways, and induction of satiety [14]. It also have repressive effect on histamine release from basophils, which indicates its potential role in the pathogenesis of disorders involving basophil degranulation including urticaria [14]. Lower levels of apo A-IV have been found to correlate with higher disease severity in allergic rhinitis [15]. Taking into consideration, the role of apo A-IV in other allergic disorders, we wanted to evaluate it in our study, as it could be a potential therapeutic target in urticaria management. With a growing interest among allergologists for the identification of a standard biomarker of disease severity and antihistamine response in CSU, we aim to assess the role of IL-9, C5a, and apo A-IV as biomarkers of disease severity and antihistamine response in CSU patients.

## 2. Materials and methods

### 2.1. Study design

Ours was a single-center-based prospective observational study conducted in urticaria clinic of a UCARE center [16], wherein CSU patients aged more than 18 years were screened for eligibility from April 2021 to November 2021 and consenting patients were recruited. Patients with inducible urticaria, hereditary angioedema, anaphylaxis, and urticarial vasculitis were excluded based on predefined criteria. The study protocol was approved by institutional ethics committee and registered in clinical trials registry of India (CTRI/2021/04/032718).

### 2.2. Objectives and outcomes

The following outcome measures were assessed:

1. Levels of serological markers (C5a, IL-9 and apo A-IV) in both CSU cases and healthy controls.
2. Correlation of C5a, IL-9, and apo A-IV levels with baseline disease severity, and comparison of levels within antihistamine responders and nonresponders.
3. Serum IgE and anti-thyroid peroxidase (TPO) antibodies were assessed in antihistamine responders and nonresponders and were correlated with baseline disease severity.

### 2.3. Assessment of disease severity

Baseline disease severity was assessed using urticaria activity score over past 7 consecutive days (UAS 7) [17]. Patients were followed up at day 15, 30, 60, and 90; and UAS 7 was assessed at each follow-up. Patients were initiated uniformly on standard dose of levocetirizine (5 mg/d), which was up dosed to a maximum of 4-fold, that is, 20 mg/d in nonresponders, as per European Academy of Allergy and Clinical Immunology (EAACI) guidelines [1]. Antihistamine nonresponders were those in whom disease was not controlled with maximum dosage of 20 mg levocetirizine permitted as per EAACI guidelines [1].

### 2.4. Evaluation of serological markers

Estimation of serum levels of IL-9 and apo A-IV was done in 95 CSU patients and 42 controls, whereas C5a levels were assessed in 82 patients and 42 controls. Absorbance was evaluated at 450 nm on a multimode reader (Tecan, AG, Switzerland).

### 2.5. Other baseline investigations

**Figure 1.**
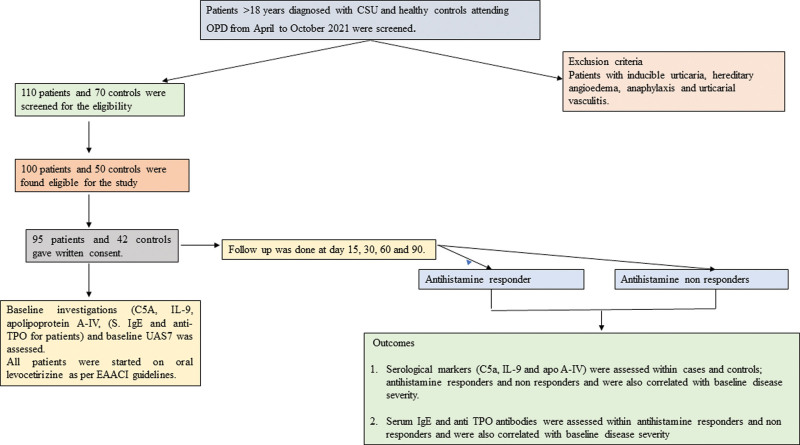
Flowchart depicting the study outline.

All CSU patients were also evaluated for total serum IgE levels and anti-TPO antibodies at baseline in addition to abovementioned serological markers at baseline. The study protocol is outlined in Fig. 1.

### 2.6. Statistical analysis

Descriptive statistics were summarized in the form of the mean (±standard deviation [SD]), median (interquartile range) for continuous variables, and frequencies and percentages for categorical variables. Group comparisons for continuously distributed data were made using an independent sample *t* test when comparing 2 groups. Serological markers were assessed within antihistamine responders and nonresponders and also within cases and controls using Wilcoxon-Mann-Whitney *U* test. The χ^2^ test was used for group comparisons for categorical data. In case the expected frequency in the contingency tables was found to be <5 for >25% of the cells, Fisher exact test was used instead. Linear correlation within serological markers and baseline disease severity was evaluated using Spearman correlation. A 2-tailed *P* value <0.05 was considered as significant. Receiver operator characteristics (ROC) curve analysis was done to assess the optimal cutoffs to distinguish cases from controls. Logistic regression was used to estimate associations with adjustments for potential confounders.

### 2.7. Ethics

Ethical approval for this study (Ethical Committee No. INT/IEC/2021/SPL-443) was provided by the Institutional Ethical Committee of Postgraduate Institute of Medical Education and Research, Chandigarh, India (Convener and Chairman, Prof. Nandita Kakkar and Prof. Sanjeev Handa, respectively) on March 12, 2021.

## 3. Results

Ninety-five consenting CSU patients and 42 healthy controls were recruited. All patients were followed up for 90 days at day 15, 30, 60, and 90. Of 95 CSU patients, 25 individuals (26.3%) were identified as nonresponsive to 4-fold dosage of levocetirizine.

### 3.1. Epidemiological and disease parameters

Forty-two age- and gender-matched controls were recruited where the mean age (SD) of CSU patients was 33.77 (8.07) years and that of controls was 35.50 (7.68) years (*P* = 0.207) and male to female ratio within both cases and controls was comparable (*P* = 0.88). Clinical and epidemiological parameters of CSU patients are summarized in Table 1.

**Table 1.** Summary of the clinicoepidemiological characteristics of patients

Characteristics	Patient’s data
Total number of patients	95
Mean age (±SD)	33.77 (±8.07)
Mean (±SD) duration of illness (months)	20.82 (±16.57)
Gender (female:male)	1.41:1
Presence of thyroid disease Hyperthyroidism Hypothyroidism	0 5
History of atopy	23
Presence of angioedema	37
History of food exacerbation	13
History of exacerbation with NSAIDs	9

NSAISs, non steroidal anti-inflammatory drugs; SD, standard deviation.

### 3.2. Serological markers in CSU patients versus healthy controls

**Figure 2.**
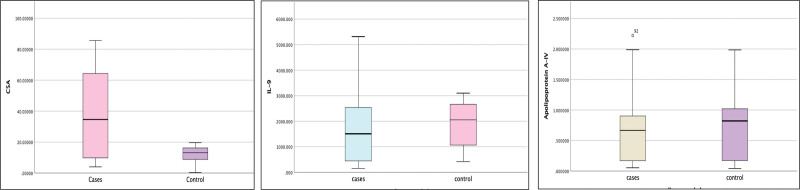
Comparison of complement fraction 5a (C5a), interleukin (IL)-9, and apolipoprotein A-IV among chronic urticaria cases and healthy controls.

Mean (SD) C5a levels were significantly higher in CSU cases 46.96 (46.72) ng/mL as compared to healthy controls 20.11 (20.24) ng/mL (*P* = 0.004). The mean (SD) of IL-9 (pg/mL) was 1,607 (1,182.5) in CSU patients and 1,838.70 (929.89) in controls. There was no significant difference in IL-9 levels within CSU patients and healthy controls (*P* = 0.082). The mean (SD) of apo A-IV (ng/L) was 0.6019 (0.4511) among CSU cases and 0.7597 (0.5589) among controls. This difference was not statistically significant (*P* = 0.173) (Table 2). Comparison of 3 serological markers within cases and controls is depicted in Fig. 2.

**Table 2.** Comparative analysis of CSU cases versus healthy controls

Parameter	Mean (SD)		Wilcoxon-Mann-Whitney *U* test	
	CSU cases	Controls	W	*P* value
Complement fraction 5a	46.96 (46.72)	20.11(20.24)	2266.000	0.004
Interleukin-9	1607(1182.5)	1838.70 (929.89)	6182.000	0.082
Apolipoprotein A-IV	0.6019(0.4511)	0.7597 (0.5589)	6263.000	0.173

CSU, chronic spontaneous urticaria; SD, standard deviation. Significant statistical values are emphasized using bold formatting.

Significant statistical values are emphasized using bold formatting.

### 3.3. Correlation of serological markers with baseline disease severity

**Figure 3.**
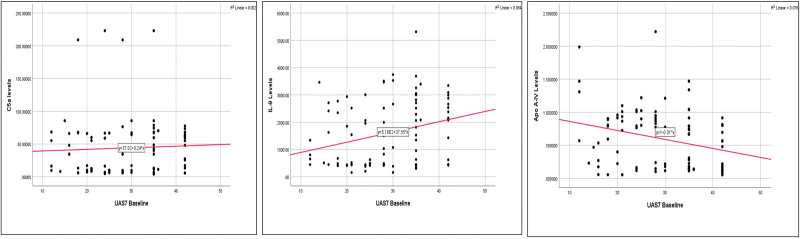
Correlation analysis between baseline urticaria activity score (UAS 7) and complement fraction 5a (C5a), interleukin (IL)-9, and apolipoprotein A-IV.

There was a weak positive correlation between baseline disease severity and C5a levels, but it was not statistically significant (*ρ* = 0.06, *P* = 0.597). A statistically significant weak positive correlation was observed between IL-9 levels and baseline disease severity (*ρ* = 0.277, *P* = 0.007). There was a statistically significant negative correlation between apo A-IV levels and baseline disease severity (*ρ* = −0.271, *P* = 0.008). Correlation of 3 serological markers with disease severity is depicted in Fig. 3.

### 3.4. Serological markers in antihistamine-responsive and nonresponsive CSU patients

We found no statistically significant difference in the levels of C5a (*P* = 0.637) and apo A-IV (*P* = 0.460) within antihistamine responders and nonresponders whereas significantly higher levels of IL-9 were observed in antihistamine responders as compared to nonresponders (*P* = 0.008) (Table 3).

**Table 3.** Comparative analysis of antihistamine responders and nonresponder subgroups

Parameter	Mean (SD)		Wilcoxon-Mann-Whitney U Test	
	Antihistamine responders	Antihistamine nonresponders	W	*P* value
Serum immunoglobulin E levels (IU/mL)	203.35 (167.22)	345.67 (291.70)	585.000	0.031
Antithyroid peroxidase antibodies (IU/L)	39.82 (165.02)	158.06 (368.69)	574.500	0.039
Complement fraction 5a	45.79 (45.01)	53.78 (57.51)	456.500	0.637
Interleukin-9	1804.00 (1191.7)	1056 (982.02)	563.500	0.008
Apolipoprotein A-IV	0.5667 (0.4469)	0.7005 (0.4570)	787.500	0.460

Significant statistical values are emphasized using bold formatting.

### 3.5. ROC curve analysis of C5a, IL-9, and apo A-IV levels

**Figure 4:**
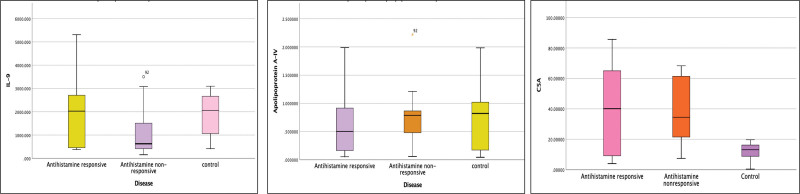
Comparison of complement fraction 5a (C5a), interleukin (IL)-9, and apolipoprotein A-IV among antihistamine responders, nonresponders, and healthy controls.

ROC curve analysis of C5a, IL-9, and apo A-IV to assess their diagnostic performance showed that C5a had the best diagnostic accuracy (Fig. 4, Table 4).

**Table 4.** ROC analysis of serological markers among various subgroups

Subgroup	Marker	Cutoff value	Area area under the receiver operating characteristic curve	95% confidence interval	*P* value	Sn	Sp	PPV	NPV	Diagnostic accuracy
Chronic spontaneous urticaria vs controls	C5a	25.64	0.658	0.559–0.757	0.004	61%	83%	88%	52%	68%
	IL-9	507.43	0.557	0.457–0.658	0.082	33%	95%	93%	42%	54%
	Apo A-IV	0.807	0.595	0.484–0.705	0.173	70%	55%	75%	48%	64%
Antihistamine responders vs controls	C5a	34.46	0.638	0.533–0.743	0.015	57%	86%	87%	54%	68%
	IL-9	507.43	0.541	0.435–0.648	0.467	33%	95%	92%	46%	56%
	Apo A-IV	0.807	0.579	0.466–0.693	0.160	67%	55%	71%	50%	62%
Antihistamine nonresponders vs controls	C5a	25.64	0.776	0.614–0.937	0.004	75%	83%	56%	92%	82%
	IL-9	520.5	0.653	0.448–0.857	0.001	42%	93%	62%	85%	82%
	Apo A-IV	0.807	0.686	0.529–0.842	0.448	83%	55%	34%	92%	61%
Antihistamine responders vs nonresponders	C5a	≤17.05	0.543	0.384–0.703	0.637	41.4%	83.3%	93.5%	19.6%	47.6%
	IL-9	1764.69	0.583	0.42–0.745	0.008	55.7%	66.7%	90.7%	20.5%	57.3%
	Apo A-IV	0.137	0.624	0.45–0.799	0.460	89.4%	41.75	89.4%	31.2%	78%

Apo A-IV, apolipoprotein A-IV; AUROC, area under the receiver operating characteristic curve; C5a, complement fraction 5a; IL-9, interleukin-9; NPV, negative predictive value; PPV, positive predictive value; ROC, receiver operating characteristic; Sn, sensitivity; Sp, specificity.

Significant statistical values are emphasized using bold formatting.

### 3.6. Correlation of levels of total IgE and anti-TPO antibodies with disease severity and antihistamine response

Elevated serum IgE levels were found in 60 (63.1%) of 95 patients. The mean (SD) of serum IgE (IU/mL) was 345.67 (291.70) in nonresponders, which was significantly higher compared to responders, that is, 203.35 (167.22) (*P* = 0.031). There was no significant correlation found within baseline disease severity and serum IgE (*ρ* = 0.07, *P* = 0.549) and anti-TPO antibody levels (*ρ* = −0.14, *P* = 0.203). Elevated anti-TPO antibody levels were noted in 12 (12.6%) patients. The mean (SD) level of anti-TPO was 158.06 (368.69) in nonresponders, which was significantly higher compared to responders, that is, 39.82 (165.02) (*P* = 0.039).

## 4. Discussion

Chronic urticaria is a distressing disease, which significantly impairs the quality of life and has profound economic burden on the patient. Despite numerous studies conducted to identify promising biomarkers for disease severity and prediction of treatment response in patients with CSU, a definitive delineation is yet to be established [18, 19]. Our study was aimed at assessing role of 3 novel markers in CSU. In addition, role of 2 commonly evaluated markers in urticaria, that is, total serum IgE and anti-TPO antibody was also assessed in predicting disease severity and response to antihistamine therapy.

In our study, 25 (26.3%) patients were found to be unresponsive even to 20 mg of levocetirizine. This finding was is in line with previous literature that suggests approximately 10%–50% of patients will not respond satisfactorily to higher than the conventional dosage of antihistamines, necessitating exploration of alternative treatment options [20]. There have been several reports with inconsistent results regarding the role of anti-TPO and serum IgE levels in assessing the antihistamine response [21, 22]. In our study, we found elevated anti-TPO antibody levels in 12 (12.6%) patients at baseline. Several reports have suggested that a significant number of CSU patients (up to 33% in few studies) shows elevated levels of autoantibodies against thyroid antigens [23–25]. We found significantly elevated levels of anti-TPO antibodies in antihistamine nonresponders as compared to responders (*P* = 0.039). These findings indicate that higher anti-TPO levels might predict nonresponse to antihistamines. As per recent literature, there are 2 subtypes of CSU including autoallergic urticaria (type I autoimmunity) that has IgE autoantibodies to self-antigens and autoimmune urticaria (type IIb autoimmunity) having mast cell-directed activating autoantibodies. Later subtype is more likely to have low or very low total IgE levels and increased levels of IgG anti-TPO antibodies. Recent EAACI guidelines indicate that a high ratio of IgG anti-TPO to total IgE is currently the best surrogate marker for type IIb autoimmune CSU [1]. Although in our study we were not able to estimate the exact subtype of anti-TPO antibodies, we found elevated serum IgE levels in 60 (63.1%) patients. There were significantly higher levels of serum IgE in antihistamine nonresponders compared to responders (*P* = 0.031). According to the previous literature, there has been conflicting reports regarding the role of serum IgE levels in predicting disease responsiveness to antihistamines [26, 27]. Although lower IgE levels are known to be an indicator of poor response to omalizumab [28], its role as a marker of antihistamine responsiveness is elusive. Findings from our study hint that elevated serum IgE levels might support the literature that its higher IgE levels can predict unresponsiveness to conventional dosage of antihistamines.

Elevation of C5a levels in CSU patients has been documented previously in various studies [3, 29]. We also found a significant elevation of C5a levels in CSU cases as compared to healthy controls (*P* = 0.004). Higher C5a levels in patients support the role of complement system activation in the development of urticaria. Role of C5a as a marker of antihistamine resistance was also assessed by Huilan et al. [30] in 40 chronic urticaria patients who observed higher plasma levels of C5a in antihistamines-resistant chronic urticaria patients than antihistamine-responsive patients. However, we did not find any significant difference in C5a levels among antihistamine-responsive and nonresponsive patients (*P* = 0.637). There was no significant correlation found within C5a levels and baseline disease severity. Similar findings were noted by Metz et al. [31] in their study where there was no significant correlation between C5a levels and disease activity.

A possible role of IL-9 in the pathogenesis chronic urticaria has been postulated by various studies [11, 12, 32]. In our study, we did not observe any significant difference in levels of IL-9 within cases and controls (*P* = 0.082). In a similar study, Metz et al. [31] reported no significant elevation in IL-9 levels in CSU patients. There were significantly higher levels of IL-9 in antihistamine responders as compared to antihistamine non responders (*P* = 0.008), indicating a possible role of IL-9 as a marker of antihistamine responsiveness. There was a weak positive correlation between baseline UAS 7 and IL-9 that was statistically significant (*P* = 0.007), which suggests that higher IL-9 levels might be an indicator of severe disease. This was contrary to the results of previous studies where no correlation between serum IL-9 levels and disease severity was observed [12, 31].

Previous studies have suggested a potential link between apo A-IV and allergic diseases, such as asthma and allergic rhinitis [14, 33, 34]. In asthma, decreased levels of apo A-IV have been reported in bronchial epithelial cells of patients, indicating a potential protective role of apo A-IV in airway inflammation [14]. Previous studies have not investigated the role of apo A-IV (apo A-IV) in patients with CSU. In our study, we observed a significant negative correlation between apo A-IV levels and baseline disease severity (*ρ* = −0.271, *P* = 0.008), indicating that lower apo A-IV levels might predict higher disease activity. This was in line with previous studies that noticed lower apo A-IV levels in active allergic diseases [14, 33]. However, we found no significant difference in apo A-IV levels between antihistamine responders and nonresponders (*P* = 0.460). The findings of our study suggest a potential role of apo A-IV as a biomarker of disease severity in CSU.

Few limitations of our study include smaller number of controls and antihistamine nonresponsive patients, single-time testing of serological markers, and single-center design. Multivariate logistic regression to find out the strength of association of statistically significant markers with disease activity could not be accomplished because of small sample size.

In conclusion, identification of a reliable biomarker for assessing disease severity and treatment response is crucial for comprehensive management of chronic urticaria. Such a biomarker would not only enhance our understanding of the pathogenesis of urticaria but also aid monitoring of disease activity, prediction of treatment efficacy, and potentially contribute to future treatment prospects. Findings from our study indicate that there might be a role of lower apo A-IV and higher IL-9 values as markers of disease severity and IL-9, anti-TPO, and serum IgE as markers of antihistamine responsiveness. Although analysis of these markers in our study did not allow us to firmly conclude that these can be introduced as in routine practice as suitable biomarkers for assessing disease severity and response to antihistamines in CSU, we do not want to rule out the possibility that in the future these mediators might find a role. Therefore, we recommend that further studies with larger sample size and more frequent evaluation of these biomarkers be conducted to enhance our understanding of their roles and significance.

## Acknowledgements

We thank Dr. Rishi Gupta and Dr. Aman Sachdeva for contributing in statistical analysis. We also thank Dr. Vinod Kumar Sharma for carrying out enzyme-linked immunosorbent assay kit analysis.

## Conflicts of interest

The authors have no financial conflicts of interest.

## Author contributions

Conception or design of the work: Muthu Sendhil Kumaran, Anuradha Bishnoi, Keshavamurthy Vinay, Davinder Parsad. Data acquisition, analysis, and interpretation: Divya Bhatia, Hitaishi Mehta, Niharika Srivastava. Drafting the manuscript: Divya Bhatia, Hitaishi Mehta. Revising manuscript critically for important intellectual content: Muthu Sendhil Kumaran, Anuradha Bishnoi, Keshavamurthy Vinay, Davinder Parsad. Final approval of the version to be published: All authors. Agreement to be accountable for all aspects of the work: Muthu Sendhil Kumaran and Divya Bhatia.
